# Factors influencing burnout, stress levels, and coping strategies among nursing staff in intensive care units

**DOI:** 10.3389/fpubh.2025.1530353

**Published:** 2025-05-22

**Authors:** Beata Wudarczyk, Sabina Krupa-Nurcek, Michał Czapla, Izabella Uchmanowicz

**Affiliations:** ^1^Department of Nursing, Institute of Health Sciences, Collegium Medicum, University of Zielona Góra, Zielona Góra, Poland; ^2^Department of Surgery, Faculty of Medicine, University of Rzeszów, Rzeszów, Poland; ^3^Division of Scientific Research and Innovation in Emergency Medical Service, Department of Emergency Medical Service, Faculty of Nursing and Midwifery, Wroclaw Medical University, Wroclaw, Poland; ^4^Group of Research in Care (GRUPAC), Faculty of Health Science, University of La Rioja, Logroño, Spain; ^5^Division of Research Methodology, Department of Nursing, Faculty of Nursing and Midwifery, Wroclaw Medical University, Wroclaw, Poland; ^6^Centre for Cardiovascular Health, Edinburgh Napier University, Edinburgh, United Kingdom

**Keywords:** nurses, burnout, professional, intensive care units, stress, psychological, coping mechanisms

## Abstract

**Introduction:**

Professional burnout among nurses, particularly in intensive care units, is a significant issue affecting both healthcare professionals and patient care quality. It contributes to increased medical errors and diminished care standards. The objective of this study was to evaluate factors influencing professional burnout in nursing staff working in intensive care units.

**Methodology:**

This quantitative study was conducted between March and November 2019 among nurses undergoing qualification and specialization training at the European Centre for Postgraduate Education in Wrocław. A total of 286 questionnaires were collected, with 282 valid responses after excluding incomplete questionnaires (1.4%). Standardized tools used included the Maslach Burnout Inventory (MBI), Perceived Stress Scale (PSS-10), and the Mini-COPE Stress Coping Inventory. Participation was anonymous and voluntary, with informed consent obtained from all respondents.

**Results:**

The study included 282 respondents, with women comprising 93.62% of the participants. The average burnout score based on the MBI was 39.78 out of 100 (SD = 20.7). According to the PSS-10, 43.26% of respondents experienced high stress, 36.88% medium stress, and 19.86% low stress. The Mini-COPE results indicated frequent use of Active Coping and Planning strategies, while strategies like Denial and Substance Use were used infrequently. Regarding life satisfaction (SWLS), 41.84% of participants had medium, 32.27% high, and 25.89% low life satisfaction.

**Conclusion:**

Professional burnout among nursing staff is a multifaceted issue closely related to stress levels, coping mechanisms, and overall life satisfaction. Addressing burnout requires comprehensive approaches that consider these interrelated factors.

**Practical implications:**

To reduce burnout among ICU nurses, healthcare institutions should integrate routine stress assessments and provide structured support systems, such as resilience training and peer support programs. These interventions can enhance nurses’ ability to manage stress, decrease emotional exhaustion, and ultimately improve the quality of care delivered to patients in high-stress environments like intensive care units.

## 1 Background

Professional work not only meets existential needs but also fosters personal development and fulfillment. The nursing profession, rooted in the desire to help others, holds a unique place in this context (1). However, the number of people pursuing nursing education and entering the profession declines each year. Young people are discouraged by low prestige, high physical and mental demands, and inadequate compensation. Additionally, the aging nursing workforce further complicates recruitment and retention efforts (2). Given the vital role of nurses in therapeutic teams and their proportion in the healthcare workforce, it is essential to understand the factors contributing to professional burnout (3). Burnout leads to various individual and societal issues, including sleep disorders, behavioral problems, and disturbances in immune, digestive, and circulatory systems. It affects personal life, reduces work quality, increases medical errors and sick leave, and contributes to staffing shortages as many nurses leave the profession. In severe cases, burnout can result in addiction, depression, or suicide (4). ICU nurses are particularly vulnerable due to the high emotional demands of their work, including constant vigilance, time-pressured decisions, and frequent exposure to critical patient conditions. Prolonged stress and insufficient coping mechanisms increase the risk of burnout. Economic pressures, staffing shortages, shift work, and sleep deprivation further exacerbate the problem, contributing to higher rates of psychosomatic illnesses such as cancer and mental health disorders. Exploring the link between burnout and psychosomatic diseases is crucial, given the premature mortality in this profession (5).

The concept of professional burnout was first introduced in 1974 by American psychiatrist Freudenberger (6), who defined it as a decrease in an individual’s energy level, resulting from the overload of dealing with other people’s problems and the excessive demands placed by the work environment. Burnout is a state of fatigue or frustration arising from devoting oneself to a cause, lifestyle, or relationship that does not yield the expected rewards. In essence, failure to achieve a goal leads to discouragement and apathy (7). Burnout is a psychosocial phenomenon that arises as a response to chronic interpersonal stressors observed in the workplace. This syndrome reflects an ongoing process.

According to Maslach, burnout involves three key elements: loss of energy, where the individual feels overwhelmed, stressed, and exhausted; loss of enthusiasm, where cynicism replaces passion, patients are perceived as burdens, there is a sense of threat from superiors, and colleagues are seen as obstacles; and loss of confidence, as the lack of engagement and motivation makes it difficult to see the positive aspects of professional activity. Skills such as expertise, creativity, and empathy diminish, and the individual enters an energy-saving mode (8, 9).

Pines and Aronson define burnout as a state of physical, emotional, and mental exhaustion caused by prolonged involvement in emotionally demanding situations (10). What distinguishes burnout from other concepts, such as job-related stress, fatigue, alienation, depression, and existential crisis, is that burnout is always the final result of a gradual disillusionment with the idea of finding meaning in professional life. According to Pines, engagement in one’s work is understood as a factor contributing to fulfillment in the profession, mental wellbeing, and possessing the necessary competencies to perform duties in the workplace (11).

From another perspective, Cherniss linked burnout with changes in motivation, describing it as withdrawal from professional activity due to excessive stress or dissatisfaction with one’s job (12). According to him, burnout is caused by prolonged stress, leading to fatigue, impulsivity, and a feeling of tension. With burnout comes a loss of enthusiasm, commitment, and a sense of purpose, eventually resulting in isolation, emotional distancing from patients, and cynical or even abrasive behavior. Burnout is the body’s response to stress caused by the work environment, particularly when the job fails to provide satisfaction, when the employee is overloaded with responsibilities, faces excessive demands, or when the work becomes monotonous and boring. Stress from overwork leads to the burnout effect. A characteristic feature of those affected by burnout is the discrepancy between feeling competent and completely ineffective. These individuals lose engagement and see no value in their actions (13).

Schaufeli and Enzmann compared burnout syndrome to a battery that gradually discharges despite being supplied with energy, as more is drawn from it than it receives (4). Engagement in work is not commensurate with what the employee receives in return. According to the researchers, burnout is “a persistent, negative state related to work that occurs in generally healthy individuals. It is primarily characterized by exhaustion, accompanied by psychological and physical discomfort, a sense of decreased efficacy, reduced motivation, and dysfunctional attitudes and behaviors at work. This state develops gradually and stems from a mismatch between professional intentions and realities. Burnout is often a self-perpetuating process due to inadequate coping strategies” (14). Stress arises from various factors, typically categorized into three main stressors: physical, social, and psychological. Physical stressors disrupt the body’s equilibrium and include changes in temperature, pressure, humidity, noise, strong light, radiation, and vibrations. Social stressors are related to group life, especially in the workplace. Sources of stress in this context may include poor communication, lack of acceptance or understanding of norms, and flawed group structures. Stress can result from the communication style and a lack of proper interaction between employees and supervisors or among co-workers (15).

Psychological sources of stress are divided into four categories: disturbances, threats, overload, and deprivation. Disturbance occurs when specific circumstances force an individual to exert more effort. A threat is a situation where there is a likelihood of an accident, bodily harm, material, or moral loss. Threat-related stress can stem from a real situation or anticipated danger. Threats may be physical or social, linked to fulfilling multiple social roles, continuous competition, or being subjected to constant evaluation by others. Overload occurs when an individual performs tasks at the limit of their physical and mental capabilities. A form of overload is discomfort, such as working in unpleasant conditions. Deprivation is a state of chronic unfulfilled needs, which can occur in situations like being in shelters, psychiatric hospitals, submarines, or polar expeditions (16).

### 1.1 Aim

The aim of this study was to assess factors influencing professional burnout among nursing staff in intensive care units (ICUs).

## 2 Methods

### 2.1 Research design

The study was conducted among a group of nurses participating in qualification and specialization training courses at the European Centre for Postgraduate Education in Wrocław. The study was anonymous and voluntary, and it took place between March and November 2019. The project was approved by the Bioethics Committee at the Wrocław Medical University (Approval No. KB-12/2019) and the Directorate of the European Centre for Postgraduate Education in Wrocław. Incomplete responses to the questionnaire or failure to meet any of the inclusion criteria resulted in the exclusion of the questionnaire from further analysis.

### 2.2 Sample size

A total of 286 questionnaires were collected, but 4 (1.4%) were excluded due to incomplete responses or failure to meet the inclusion criteria. Eleven individuals did not consent to participate in the study. The participants were informed about the purpose, assumptions, and course of the study. All respondents provided informed consent to participate.

### 2.3 Recruitment

The following inclusion criteria were established for recruitment to the study: possessing a valid nursing license; current employment in an intensive care unit (ICU); at least 2 years of work experience; and consent to participate in the study.

### 2.4 Outcome measures

#### 2.4.1 Demographic and work-related data questionnaire

The demographic section consisted of 9 questions and allowed for the collection of variables related to sociodemographic characteristics such as sex, age, education (including postgraduate), marital status, and place of residence. In terms of work-related characteristics, the questionnaire gathered information on years of experience in the profession, years of experience in an intensive care unit (ICU), and work system.

#### 2.4.2 Maslach burnout inventory (MBI)

The Maslach Burnout Inventory (MBI), developed by Christina Maslach (17), in its Polish adaptation (18), allows for the assessment of three dimensions of professional burnout: emotional exhaustion, depersonalization, and personal achievement. The questionnaire consists of 22 statements across three subscales: 9 statements on emotional exhaustion, 5 on depersonalization, and 8 on reduced personal achievement. Respondents rated each statement on a four-point scale assessing the frequency of their feelings: 1 – very often, 2 – sometimes, 3 – rarely, 4 – never. Results for each subscale are expressed on a scale from 0 to 100, where higher scores indicate higher levels of burnout. Additionally, a general burnout index is calculated as the average score from the three subscales. This questionnaire is the most commonly used tool for assessing professional burnout (19).

#### 2.4.3 Mini-COPE inventory for coping with stress

The Mini-COPE Inventory is used to assess typical ways of reacting and coping in situations of severe stress. The inventory consists of 28 statements divided into 14 stress-coping strategies (with two statements per strategy). The Polish version was developed by Juczyński and Ogińska-Bulik (20). The coping strategies include Active Coping, Planning, Positive Reappraisal, Acceptance, Sense of Humor, Turning to Religion, Seeking Emotional Support, Seeking Instrumental Support, Engagement in Distraction Activities, Denial, Venting, Substance Use, Behavioral Disengagement, and Self-Blame. Responses are rated on a scale from 0 to 3 points: 0 – I almost never act this way, 1 – I rarely act this way, 2 – I often act this way, 3 – I almost always act this way.

#### 2.4.4 Perceived stress scale (PSS-10)

The Perceived Stress Scale (PSS-10) was originally developed by Cohen, Kamarck, and Mermelstein (21), and the Polish adaptation was created by Juczyński and Ogińska-Bulik (20). The scale consists of 10 questions related to subjective feelings about personal problems, behaviors, and ways of coping with stress. Respondents answered each question using a five-point scale assessing thoughts and feelings experienced in the last month: 0 – never, 1 – almost never, 2 – sometimes, 3 – fairly often, 4 – very often.

### 2.5 Statistical analysis

Quantitative variables (i.e., those expressed numerically) were analyzed by calculating the mean, standard deviation, median, quartiles, minimum, and maximum values. Qualitative variables (i.e., those not expressed numerically) were analyzed by calculating the number and percentage of occurrences of each value. The Mann–Whitney test was used to compare the values of quantitative variables between two groups. The Kruskal-Wallis test was applied for comparisons across three or more groups. Post-hoc analysis was performed using Dunn’s test to identify statistically significant differences between groups when statistically significant differences were detected. Correlations between quantitative variables were analyzed using Spearman’s correlation coefficient. Multivariate analysis of the independent influence of multiple variables on a quantitative variable was performed using linear regression. The results were presented as regression model parameter values with a 95% confidence interval. A significance level of 0.05 was assumed for the analysis, meaning that all *p*-values below 0.05 were interpreted as indicating statistically significant relationships. The analysis was conducted using R software, version 3.6.2.

### 2.6 Ethics statement

The study was conducted in accordance with the Declaration of Helsinki and approved by the Ethics Committee of Wrocław Medical University no. KB-12/2019.

## 3 Results

### 3.1 Participants

The study group consisted of 282 individuals. The average age was 42.24 years (SD = 9.62), ranging from 24 to 60 years. The largest group was in the 41–50 age range (42.91%). Women dominated the group, representing 93.62% of respondents. The distribution of educational levels (secondary, bachelor’s, and master’s) was comparable. A slightly larger group had a master’s degree (35.82%). The most common form of postgraduate education was qualification courses, held by 70.57% of respondents. Slightly fewer (63.12%) had completed specialist courses, and more than half had completed a specialization. Nearly one in 10 respondents (9.57%) had completed other forms of postgraduate education. The average length of professional experience was 19.97 years (SD = 10.45), ranging from 2 to 40 years. The largest group, over one-third of the respondents, had 21–30 years of work experience. The average length of experience in an ICU was 14.26 years (SD = 9.24), ranging from 2 to 40 years, with most nurses (40.43%) having up to 10 years of ICU experience. The majority of respondents (83.69%) reported working in a shift system. Marital status included both partnered and single individuals, with nearly three-quarters of respondents being in a relationship. Over three-quarters of the respondents lived in a city. Detailed results are presented in Supplementary Table S1.

### 3.2 Results of the MBI questionnaire

The MBI assesses the level of professional burnout in three dimensions (subscales): emotional exhaustion, depersonalization, and lack of personal achievement. Scores for each subscale are expressed on a scale of 0–100, where higher scores indicate a higher level of burnout. Additionally, a general burnout index is calculated as the average of the three subscales. There are no standardized norms to determine whether burnout is severe among respondents. The overall burnout index averaged 39.78 points out of a possible 100 (SD = 20.7), with a range from 0 to 93.33 points (detailed results are presented in Supplementary Table S1).

Burnout among respondents was most strongly associated with emotional exhaustion (mean score of 53.03 points), followed by depersonalization (mean score of 39.79 points), and to a lesser extent, a lack of personal achievement (mean score of 26.51 points). Correlations between years of professional experience and work in the ICU with the level of burnout are presented in Table 1, and the analysis of sex and education in relation to the level of professional burnout is presented in Table 2 (detailed subscale results are available in Supplementary Table S2).

**Table 1:** Correlations between job tenure in the profession and work in the ICU with the level of burnout.

MBI	Correlation coefficient (Spearman)
Correlation between age and the level of burnout	
Overall MBI score	r = 0.137, *p* = 0.022*
Emotional exhaustion	r = 0.208, *p* < 0.001
Depersonalization	r = 0.171, *p* = 0.004*
Lack of professional accomplishment	r = −0.094, *p* = 0.117
Correlation between job tenure in the profession and the level of burnout	
Overall MBI score	r = 0.11, *p* = 0.065
Emotional exhaustion	r = 0.183, *p* = 0.002*
Depersonalization	r = 0.142, *p* = 0.017*
Lack of professional accomplishment	r = −0.099, *p* = 0.096
Correlation between job tenure in the ICU and the level of burnout	
Overall MBI score	r = 0.105, *p* = 0.079
Emotional exhaustion	r = 0.166, *p* = 0.005*
Depersonalization	r = 0.06, *p* = 0.004*
Lack of professional accomplishment	r = −0.008, *p* = 0.892

*Statistically significant relationship (*p* < 0.05).

**Table 2:** Analysis of sex and education in relation to the level of occupational burnout.

Sex differences in burnout levels				
MBI		Female (*N* = 264)	Male (*N* = 18)	*p*-value
Sex				
Overall MBI score	Mean ± Standard deviation	39.81 ± 20.27	39.31 ± 27.01	*p* = 0.961
	Median	38.29	43.29	
	Quartiles	26.11–54.03	17.22–65.86	
Emotional exhaustion	Mean ± Standard deviation	53.32 ± 31.07	48.77 ± 40	*p* = 0.597
	Median	55.56	44.44	
	Quartiles	30.56–77.78	11.11–86.11	
Depersonalization	Mean ± Standard deviation	39.39 ± 27.57	45.56 ± 32.03	*p* = 0.461
	Median	40	40	
	Quartiles	20–60	20–75	
Lack of professional accomplishment	Mean ± Standard deviation	26.7 ± 25.16	23.61 ± 28.4	*p* = 0.444
	Median	25	25	
	Quartiles	0–50	0–34.38	

Educational differences in burnout levels					
Education level		Secondary nursing education - a (*N* = 93)	Bachelor’s degree in nursing - B (*N* = 88)	Master’s degree in nursing - C (*N* = 101)	*p*-value
Overall MBI score	Mean ± SD	42.07 ± 21.54	42.65 ± 21.55	35.16 ± 18.44	*p* = 0.036* B, A > C
	Median	39.26	42.5	35.65	
	Quartiles	27.41–57.5	26.11–55.93	23.61–47.69	
Emotional exhaustion	Mean ± SD	56.03 ± 31.68	56.94 ± 32.83	46.86 ± 29.88	*p* = 0.046* B, A > C
	Median	55.56	55.56	44.44	
	Quartiles	33.33–77.78	30.56–88.89	22.22–66.67	
Depersonalization	Mean ± SD	44.52 ± 28.15	43.86 ± 28.75	31.88 ± 25.17	*p* = 0.001* A, B > C
	Median	40	40	20	
	Quartiles	20–60	20–60	20–40	
Lack of professional accomplishment	Mean ± SD	25.67 ± 25.36	27.13 ± 26.07	26.73 ± 24.88	*p* = 0.946
	Median	25	25	25	
	Quartiles	0–37.5	0–50	0–50	

*N*, number of participants; SD, standard deviation; *statistically significant relationship (*p* < 0.05).

### 3.3 Results of the PSS-10 questionnaire

The PSS-10 questionnaire allows for the assessment of the severity of perceived stress. For the PSS-10, sten norms are available, enabling the interpretation of raw scores. Typically, sten scores of 5 and 6 indicate average stress levels, sten scores from 7 to 10 represent high stress levels, and sten scores from 1 to 4 reflect low stress levels. Out of 282 participants, 122 (43.26%) reported high stress levels, 104 (36.88%) had moderate stress levels, and 56 (19.86%) reported low stress levels. The data are presented in the Supplementary Table S3.

### 3.4 Results of the mini-COPE questionnaire

The Mini-COPE questionnaire assesses the frequency of using 14 stress-coping strategies. Each strategy is represented by two questions in the questionnaire, for which an average score is calculated. The frequency of each strategy is expressed on a scale from 0 to 3. The strategies Active Coping and Planning were used between “often” and “almost always” (average between 2 and 3). The strategies Positive Reappraisal, Acceptance, Turning to Religion, Seeking Emotional Support, Seeking Instrumental Support, Engagement in Distraction Activities, Venting, and Self-Blame were used between “rarely” and “often” (average between 1 and 2). The strategies Sense of Humor, Denial, Substance Use, and Behavioral Disengagement were used between “almost never” and “rarely” (average between 0 and 1). The data are presented in Table 3.

**Table 3:** Results of the mini-COPE questionnaire analysis.

Strategy	*N*	Mean	SD	Median	Min	Max	Q1	Q3
Active coping	282	2.13	0.63	2	0	3	2	2.5
Planning	282	2.12	0.56	2	0	3	2	2.5
Positive reinterpretation	282	1.85	0.68	2	0	3	1.5	2
Acceptance	282	1.83	0.65	2	0	3	1.5	2
Humor	282	0.91	0.6	1	0	3	0.5	1.5
Turning to religion	282	1.12	0.9	1	0	3	0	2
Seeking emotional support	282	1.85	0.77	2	0	3	1.5	2.5
Seeking instrumental support	282	1.81	0.73	2	0	3	1.5	2.5
Self-distraction	282	1.62	0.68	1.5	0	3	1	2
Denial	282	0.92	0.72	1	0	3	0.5	1.5
Venting	282	1.35	0.63	1.5	0	3	1	2
Substance use	282	0.29	0.52	0	0	2.5	0	0.5
Behavioral disengagement	282	0.75	0.6	1	0	3	0	1
Self-blame	282	1.26	0.76	1	0	3	1	2

*N*, number of participants; SD, standard deviation; Min, minimum; Max, maximum; Q1, first quartile; Q3, third quartile.

### 3.5 Correlations between PSS-10 and MBI questionnaire result

The PSS-10 shows a statistically significant (*p* < 0.05) and positive (*r* > 0) correlation with the overall MBI score, emotional exhaustion, depersonalization, and lack of personal achievement. This indicates that the higher the level of perceived stress, the greater the degree of professional burnout in these areas. The relationship between PSS-10 and the level of professional burnout is presented in Table 4.

**Table 4:** The relationship between PSS-10 and the level of professional burnout.

MBI	PSS-10
Overall MBI score	r = 0.409, *p* < 0.001*
Emotional exhaustion	r = 0.346, *p* < 0.001*
Depersonalization	r = 0.19, *p* = 0.001*
Lack of professional accomplishment	r = 0.369, *p* < 0.001*

*Statistically significant relationship (*p* < 0.05).

### 3.6 Correlations between mini-COPE and MBI questionnaire result

Active Coping showed a statistically significant (*p* < 0.05) and negative (*r* < 0) correlation with the overall MBI score, emotional exhaustion, and lack of personal achievement, indicating that more frequent use of this strategy was associated with lower levels of professional burnout. Similarly, Planning demonstrated statistically significant negative correlations with the overall MBI score, emotional exhaustion, depersonalization, and lack of personal achievement, suggesting that those who employed this strategy more often experienced lower burnout.

Positive Reappraisal and Acceptance also correlated statistically significantly (*p* < 0.05) and negatively (*r* < 0) with the overall MBI score, emotional exhaustion, depersonalization, and lack of personal achievement, reinforcing the protective effect of these strategies against burnout.

No statistically significant correlations were observed for Sense of Humor and Seeking Instrumental Support (all *p* > 0.05).

Turning to Religion was statistically significantly (*p* < 0.05) and negatively (*r* < 0) correlated with depersonalization, indicating that more frequent use of this strategy was linked to lower levels of depersonalization.

Seeking Emotional Support showed statistically significant (*p* < 0.05) and negative (*r* < 0) correlations with the overall MBI score, depersonalization, and lack of personal achievement, suggesting that those seeking emotional support experienced lower burnout in these areas.

In contrast, Denial correlated statistically significantly (*p* < 0.05) and positively (*r* > 0) with the overall MBI score, emotional exhaustion, and lack of personal achievement, indicating that more frequent use of this strategy was associated with higher levels of burnout.

Venting showed a mixed pattern, with statistically significant (*p* < 0.05) negative correlations with depersonalization and statistically significant positive correlations with lack of personal achievement, suggesting that while it may reduce depersonalization, it could contribute to a sense of professional dissatisfaction.

Substance use, behavioral disengagement, and self-blame were all statistically significantly (*p* < 0.05) and positively (*r* > 0) correlated with the overall MBI score, emotional exhaustion, depersonalization, and lack of personal achievement, meaning that more frequent use of these strategies was associated with higher levels of burnout.

In summary, the statistically significant correlations between Mini-COPE and MBI scores indicated that the strategies Active Coping, Planning, Positive Reappraisal, Acceptance, and Seeking Emotional Support were associated with lower burnout levels. In contrast, denial, substance use, behavioral disengagement, and self-blame were linked to higher levels of burnout. The data are presented in Table 5.

**Table 5:** Correlations between mini COPE results and MBI results.

MBI	Spearman’s correlation coefficient
Correlation between active coping and the level of burnout	
Overall MBI score	r = −0.267, *p* < 0.001*
Emotional exhaustion	r = −0.163, *p* = 0.006*
Depersonalization	r = −0.094, *p* = 0.117
Lack of professional accomplishment	r = −0.342, *p* < 0.001*
Correlation between planning and the level of burnout	
Overall MBI score	r = −0.254, *p* < 0.001*
Emotional exhaustion	r = −0.184, *p* = 0.002*
Depersonalization	r = −0.149, *p* = 0.012*
Lack of professional accomplishment	r = −0.234, *p* < 0.001*
Correlation between positive reinterpretation and the level of burnout	
Overall MBI score	r = −0.226, *p* < 0.001*
Emotional exhaustion	r = −0.147, *p* = 0.013*
Depersonalization	r = −0.16, *p* = 0.007*
Lack of professional accomplishment	r = −0.227, *p* < 0.001*
Correlation between acceptance and the level of burnout	
Overall MBI score	r = −0.237, *p* < 0.001*
Emotional exhaustion	r = −0.19, *p* = 0.001*
Depersonalization	r = −0.178, *p* = 0.003*
Lack of professional accomplishment	r = −0.173, *p* = 0.004*
Correlation between turning to religion and the level of burnout	
Overall MBI score	r = −0.089, *p* = 0.137
Emotional exhaustion	r = −0.002, *p* = 0.974
Depersonalization	r = −0.126, *p* = 0.035*
Lack of professional accomplishment	r = −0.108, *p* = 0.069
Correlation between seeking emotional support and the level of burnout	
Overall MBI score	r = −0.168, *p* = 0.005*
Emotional exhaustion	r = −0.093, *p* = 0.118
Depersonalization	r = −0.159, *p* = 0.007*
Lack of professional accomplishment	r = −0.128, *p* = 0.031*
Correlation between denial and the level of burnout	
Overall MBI score	r = 0.007, *p* = 0.904
Emotional exhaustion	r = 0.004, *p* = 0.946
Depersonalization	r = −0.131, *p* = 0.027*
Lack of professional accomplishment	r = 0.13, *p* = 0.029*
Correlation between venting and the level of burnout	
Overall MBI score	r = 0.185, *p* = 0.002*
Emotional exhaustion	r = 0.128, *p* = 0.032*
Depersonalization	r = 0.093, *p* = 0.118
Lack of professional accomplishment	r = 0.166, *p* = 0.005*
Correlation between behavioral disengagement and the level of burnout	
Overall MBI score	r = 0.35, *p* < 0.001*
Emotional exhaustion	r = 0.231, *p* < 0.001*
Depersonalization	r = 0.12, *p* = 0.045*
Lack of professional accomplishment	r = 0.415, *p* < 0.001*
Correlation between self-blame and the level of burnout	
Overall MBI score	r = 0.206, *p* < 0.001*
Emotional exhaustion	r = 0.196, *p* = 0.001*
Depersonalization	r = 0.096, *p* = 0.107
Lack of professional accomplishment	r = 0.182, *p* = 0.002*

*Statistically significant relationship (*p* < 0.05).

## 4 Discussion

The conducted study demonstrated that age statistically significantly influences the level of burnout among nurses working in intensive care units (ICUs). The older the nurse, the greater the emotional exhaustion and depersonalization. Age is an important factor in the development of mental disorders due to a decreased ability to adapt to stressful work conditions (22). In the study by Liao et al. (23) older nurses had a higher burnout index compared to younger ones. However, researchers conducting a study in Brazil reached different conclusions, showing that nurses with <5 years of experience experienced burnout more frequently. Younger and less experienced nurses were more prone to burnout, often feeling unprepared for their professional duties (24). In addition to age, another statistically significant factor in burnout is years of work experience, both in the profession and in ICUs. Our study revealed that professional experience correlates with burnout—the longer the work experience, the higher the levels of emotional exhaustion and depersonalization. Similar conclusions were drawn by other authors (25–28), confirming that older nurses with longer work experience more frequently experience burnout syndrome. Other authors (29) found no statistically significant relationship between burnout and years of work experience. Our study confirmed that ICU work experience statistically significantly correlated with emotional exhaustion levels. In the other study (30) nurses with longer experience in anesthesiology and intensive care reported lower job satisfaction than those with shorter tenures. Wieder-Huszla et al. (31) found that nursing staff exhibited symptoms of burnout across all three dimensions, with depersonalization being more prominent in nurses with shorter work experience. Studies by foreign authors have demonstrated statistically significant correlations between age, work experience, and burnout (32–40).

The available literature provides conflicting data on this topic. Some studies confirm that older age and longer work experience are associated with higher burnout levels, while others suggest the opposite — younger individuals with less experience higher burnout. Age and work experience among ICU nurses remain an open issue, requiring further research. In our study, sex was not found to have a statistically significant effect on burnout, which aligns with the findings of other authors (41, 42). Other authors (43) found that education level and the number of completed training courses increased feelings of personal accomplishment and reduced emotional exhaustion. Similar conclusions were drawn in our study, where respondents with higher education exhibited lower levels of burnout. However, in the other research (44), authors reported different results, showing that higher education among nurses was associated with increased burnout. Another variable considered in the study was the work schedule. Our findings showed no statistically significant impact of this variable on burnout levels, differing from Boateng et al. (45), who indicated that frequent night shifts contributed to burnout. The study by Salem and Ebrahem (46) demonstrated that shift work, weekend work, and long working hours worsened burnout, with night shifts and aging being contributing factors to burnout among ICU nurses in Spain. In the RN4CAST project, Dall’Ora et al. (47) found that nurses working 12-h shifts or longer experienced burnout more frequently compared to those working 8-h shifts or less. Moreover, job satisfaction decreased as shift length increased. This apparent paradox — where nurses prefer 12-h shifts but experience lower job satisfaction — may be explained by the cumulative negative effects of long shifts, which nurses may not be fully aware of or attribute to shift work.

One of the conclusions of the study by other authors (48) is that shift work can cause hormonal disturbances and decreased sexual activity, though age was not a determinant among the nurses surveyed. In our study, we found no statistically significant influence of residence location or marital status on burnout levels. Similar findings were reported by other authors (27, 30). However, the study by Cañadas-De la Fuente et al. (49) showed that being single or divorced contributed to higher levels of burnout. A study conducted in Nigeria found that sociodemographic factors were not statistically significantly related to burnout among nurses (50).

Nursing staff working in ICUs are at risk of experiencing burnout, which can lower the quality of patient care (30). In our study, the overall burnout index averaged 39.78 points out of 100. Emotional exhaustion was the main contributor to burnout among nurses (53.03 points), followed by depersonalization (39.79 points), with lack of personal achievement being the lowest contributor (26.51 points). As early as 2007, Chmura et al. (51) reported that ICU nurses most frequently reported experiencing burnout. Freeborn (52), in their study of burnout among nurses, also found relatively high levels of burnout among ICU nurses.

### 4.1 Study limitation

One limitation of this study is its reliance on self-reported data, which may be subject to bias, as respondents may not always provide accurate assessments of their levels of stress, coping mechanisms, or burnout. Additionally, the cross-sectional design of the study captures data at a single point in time, limiting the ability to assess the progression or changes in burnout and stress levels over time. A longitudinal approach would provide more insights into how burnout develops and evolves, especially in the context of ICU nursing. Another limitation is the specific focus on nurses from intensive care units who were attending qualification and specialization courses, which may not be fully representative of the broader nursing population in other settings. This sample may have a particular set of experiences or characteristics that differ from nurses in other areas of healthcare. Furthermore, while the study examines several sociodemographic and work-related factors, it does not account for other potential variables that could influence burnout, such as personal life stressors, social support systems, or organizational factors like staffing levels and management styles. These unmeasured factors could contribute to burnout and might provide a more comprehensive understanding of its determinants. Lastly, the study was conducted within a specific geographical and cultural context, which may limit the generalizability of the findings to other regions or countries. Differences in healthcare systems, work conditions, and cultural perceptions of burnout could affect the extent to which the results apply in other settings.

## 5 Conclusion

The study highlights that emotional exhaustion is the most significant contributor to burnout among ICU nurses, followed by depersonalization and lack of job satisfaction. Key sociodemographic factors influencing burnout include older age, longer work experience, and higher education levels. Nurses with longer tenures in ICUs tend to experience higher levels of burnout, particularly in emotional exhaustion and depersonalization. Additionally, increased perceived stress levels correlate with higher burnout. However, nurses who employ constructive stress-coping strategies exhibit lower levels of burnout.

Given these findings, healthcare institutions should prioritize the implementation of targeted stress management and coping strategy training programs for ICU nurses, particularly for those with longer work experience and higher stress levels. Such interventions could help reduce emotional exhaustion and prevent burnout, thereby improving both nurse wellbeing and the quality of patient care.

## Data Availability

The raw data supporting the conclusions of this article will be made available by the authors, without undue reservation.

## References

[1] Riley JM Beal JA Ponte PR. The exemplary practice life of the nurse. J Prof Nurs. (2021) 37:1018–25. doi: 10.1016/j.profnurs.2021.07.003, PMID: 34742505 PMC8435935

[2] Marcinowicz L Owlasiuk A Slusarska B Zarzycka D Pawlikowska T. Choice and perception of the nursing profession from the perspective of polish nursing students: a focus group study. BMC Med Educ. (2016) 16:243. doi: 10.1186/s12909-016-0765-3, PMID: 27644123 PMC5029103

[3] Shah MK Gandrakota N Cimiotti JP. Prevalence of and factors associated with nurse burnout in the US. JAMA Netw Open. (2021) 4:2–11. doi: 10.1001/jamanetworkopen.2020.36469, PMID: 33538823 PMC7862989

[4] Membrive-Jiménez MJ Gómez-Urquiza JL Suleiman-Martos N Velando-Soriano A Ariza T de la Fuente-Solana EI. Relation between burnout and sleep problems in nurses: a systematic review with Meta-analysis. Healthcare. (2022) 10:954. doi: 10.3390/healthcare10050954, PMID: 35628091 PMC9140410

[5] Saravanan P Nisar T Zhang Q Masud F Sasangohar F. Occupational stress and burnout among intensive care unit nurses during the pandemic: a prospective longitudinal study of nurses in COVID and non-COVID units. Front Psych. (2023) 14:1129268. doi: 10.3389/fpsyt.2023.1129268, PMID: 36993929 PMC10040835

[6] Kwon JE. The impact of career plateau on job burnout in the COVID-19 pandemic: a moderating role of regulatory focus. Int J Environ Res Public Health. (2022) 19:1087. doi: 10.3390/ijerph19031087, PMID: 35162110 PMC8834611

[7] Hillert A Albrecht A Voderholzer U. The burnout phenomenon: a Résumé after more than 15,000 scientific publications. Front Psych. (2020) 11:519237. doi: 10.3389/fpsyt.2020.519237, PMID: 33424648 PMC7793987

[8] Ferreira NN Lucca SR. Burnout syndrome in nursing assistants of a public hospital in the state of Sao Paulo. Rev Bras Epidemiol. (2015) 18:68–79. doi: 10.1590/1980-5497201500010006, PMID: 25651012

[9] Edú-Valsania S Laguía A Moriano JA. Burnout: a review of theory and measurement. Int J Environ Res Public Health. (2022) 19:1780. doi: 10.3390/ijerph19031780, PMID: 35162802 PMC8834764

[10] Liu C-E Hu C Xie W Liu T He W. The moderated-mediation effect of workplace anxiety and regulatory focus in the relationship between work-related identity discrepancy and employee innovation. Int J Environ Res Public Health. (2020) 17:6121. doi: 10.3390/ijerph17176121, PMID: 32842458 PMC7503295

[11] Ramírez-Elvira S Romero-Béjar JL Suleiman-Martos N Gómez-Urquiza JL Monsalve-Reyes C Cañadas-De la Fuente GA. Prevalence, risk factors and burnout levels in intensive care unit nurses: a systematic review and meta-analysis. Int J Environ Res Public Health. (2021) 18:1432. doi: 10.3390/ijerph182111432, PMID: 34769948 PMC8583312

[12] Dall’Ora C Ball J Reinius M Griffiths P. Burnout in nursing: a theoretical review. Hum Resour Health. (2020) 18:41. doi: 10.1186/s12960-020-00469-9, PMID: 32503559 PMC7273381

[13] Sullivan D White KM Frazer C. Factors associated with burnout in the United States versus international nurses. Nurs Clin North Am. (2022) 57:29–51. doi: 10.1016/j.cnur.2021.11.003, PMID: 35236607

[14] Sohrabi Y Yarmohammadi H Pouya AB Arefi MF Hassanipour S Poursadeqiyan M. Prevalence of job burnout in Iranian nurses: a systematic review and meta-analysis. Work. (2022) 73:937–43. doi: 10.3233/WOR-210283, PMID: 35988237

[15] Wood RE Brown RE Kinser PA. The connection between loneliness and burnout in nurses: an integrative review. Appl Nurs Res. (2022) 66:151609. doi: 10.1016/j.apnr.2022.151609 35840269

[16] Chen YC Guo YL Chin WS Cheng NY Ho JJ Shiao JS. Patient-nurse ratio is related to Nurses’ intention to leave their job through mediating factors of burnout and job dissatisfaction. Int J Environ Res Public Health. (2019) 16:4801. doi: 10.3390/ijerph16234801, PMID: 31795420 PMC6926757

[17] Kleijweg JHM Verbraak MJPM Van Dijk M. The clinical utility of the Maslach burnout inventory in a clinical population. Psychol Assess. (2013) 25:435–41. doi: 10.1037/a0031334, PMID: 23356679

[18] Khamisa N Oldenburg B Peltzer K Ilic D. Work related stress, burnout, job satisfaction and general health of nurses. Int J Environ Res Public Health. (2015) 12:652–66. doi: 10.3390/ijerph120100652, PMID: 25588157 PMC4306884

[19] Harrad R Sulla F. Factors associated with and impact of burnout in nursing and residential home care workers for the elderly. Acta Biomed. (2018) 89:60–9. doi: 10.23750/abm.v89i7-S.7830 PMC6502144 30539935

[20] Juczyński Z Ogińska-Bulik N. Narzędzia pomiaru stresu i radzenia sobie ze stresem. Warszawa: Pracownia Testów Psychologicznych Polskiego Towarzystwa Psychologicznego (2009).

[21] Amini K Miyanaji H Din MM. Bullying and burnout in critical care nurses: a cross-sectional descriptive study. Nurs Crit Care. (2023) 28:202–10. doi: 10.1111/nicc.12744, PMID: 35146848

[22] França FM Ferrari R. Burnout syndrome and the socio-demographic aspects of nursing professionals. Acta Paul Enferm. (2012) 25:743–8. doi: 10.1590/S0103-21002012000500015

[23] Liao RW Yeh ML Lin KC Wang KY. A hierarchical model of occupational burnout in nurses associated with job-induced stress, self-concept, and work environment. J Nurs Res. (2020) 28:e79. doi: 10.1097/JNR.0000000000000348 31633639

[24] Vasconcelos EM De Martino MMF. Predictors of burnout syndrome in intensive care nurses. Rev Gaúcha Enferm. (2018) 38:e65354. doi: 10.1590/1983-1447.2017.04.65354, PMID: 29933422

[25] Fan Y Cao M Zhou Y Duan P Xing L. Relationship between workplace bullying, spirituality, and job burnout in paediatric nurses: a cross-sectional study. Nurs Open. (2023) 10:3872–80. doi: 10.1002/nop2.1645, PMID: 36790919 PMC10170933

[26] Botha E Gwin T Purpora C. The effectiveness of mindfulness based programs in reducing stress experienced by nurses in adult hospital settings: a systematic review of quantitative evidence protocol. JBI Database System Rev Implement Rep. (2015) 13:21–9. doi: 10.11124/jbisrir-2015-2380, PMID: 26571279

[27] McCleery E Christensen V Peterson K Humphrey L Helfand M. Evidence brief: The quality of care provided by advanced practice nurses. Washington, DC: Department of Veterans Affairs (US) (2014). 27606392

[28] Friganović A Selič P Ilić B Sedić B. Stress and burnout syndrome and their associations with coping and job satisfaction in critical care nurses: a literature review. Psychiatr Danub. (2019) 31:21–31. 30946714

[29] Quesada-Puga C Izquierdo-Espin FJ Membrive-Jiménez MJ Aguayo-Estremera R Cañadas-De La Fuente GA Romero-Béjar JL. Job satisfaction and burnout syndrome among intensive-care unit nurses: a systematic review and meta-analysis. Intensive Crit Care Nurs. (2024) 82:103660. doi: 10.1016/j.iccn.2024.103660, PMID: 38394983

[30] Khatatbeh H Pakai A Al-Dwaikat T Onchonga D Amer F Prémusz V. Nurses’ burnout and quality of life: a systematic review and critical analysis of measures used. Nurs Open. (2022) 9:1564–74. doi: 10.1002/nop2.936, PMID: 33991408 PMC8994939

[31] Wieder-Huszla S Żak B Jurczak A. Wypalenie zawodowe wśród personelu pielęgniarskiego. Fam Med Prim Care Rev. (2016) 1:63–8. doi: 10.5114/fmpcr/59057

[32] Gómez-Urquiza J Aneas-López A Fuente-Solana E. Prevalence, risk factors, and levels of burnout among oncology nurses: a systematic review. Oncol Nurs Forum. (2016) 43:E104–20. doi: 10.1188/16.ONF.E104-E120 27105202

[33] Duarte J Pinto-Gouveia J. The role of psychological factors in oncology nurses’ burnout and compassion fatigue symptoms. Eur J Oncol Nurs. (2017) 28:114–21. doi: 10.1016/j.ejon.2017.04.002, PMID: 28478848

[34] Gama G Barbosa F Vieira M. Personal determinants of nurses’ burnout in end of life care. Eur J Oncol Nurs. (2014) 18:527–33. doi: 10.1016/j.ejon.2014.04.005, PMID: 24888265

[35] Yu H Jiang A Shen J. Prevalence and predictors of compassion fatigue, burnout and compassion satisfaction among oncology nurses: a cross-sectional survey. Int J Nurs Stud. (2016) 57:28–38. doi: 10.1016/j.ijnurstu.2016.01.012, PMID: 27045562

[36] Taleghani F Ashouri E Saburi M. Empathy, burnout, demographic variables and their relationships in oncology nurses. Iran J Nurs Midwifery Res. (2017) 22:41–5. doi: 10.4103/ijnmr.IJNMR_66_16, PMID: 28382057 PMC5364751

[37] Eelen S Bauwens S Baillon C. The prevalence of burnout among oncology professionals: oncologists are at risk of developing burnout. Psychooncology. (2014) 23:1415–22. doi: 10.1002/pon.3579, PMID: 24846818

[38] Davis S Lind BK Sorensen C. A comparison of burnout among oncology nurses working in adult and pediatric inpatient and outpatient settings. Oncol Nurs Forum. (2013) 40:E303–11. doi: 10.1188/13.ONF.E303-E311 23803274

[39] Ko W Kiser-Larson N. Stress levels of nurses in oncology outpatient units. Clin J Oncol Nurs. (2016) 20:158–64. doi: 10.1188/16.CJON.158-164, PMID: 26991708

[40] Font A Corti V Berger R. Burnout in healthcare professionals in oncology. Procedia Econ Financ. (2015) 23:228–32. doi: 10.1016/S2212-5671(15)00320-2

[41] Wang J Okoli CTC He H Feng F Li J Zhuang L. Factors associated with compassion satisfaction, burnout, and secondary traumatic stress among Chinese nurses in tertiary hospitals: a cross-sectional study. Int J Nurs Stud. (2020) 102:103472. doi: 10.1016/j.ijnurstu.2019.103472, PMID: 31810017

[42] De la Fuente-Solana EI Pradas-Hernández L González-Fernández CT Velando-Soriano A Martos-Cabrera MB Gómez-Urquiza JL. Burnout syndrome in Paediatric nurses: a multi-Centre study. Int J Environ Res Public Health. (2021) 18:1324. doi: 10.3390/ijerph18031324, PMID: 33535707 PMC7908244

[43] Edwards-Maddox S. Burnout and impostor phenomenon in nursing and newly licensed registered nurses: a scoping review. J Clin Nurs. (2023) 32:653–65. doi: 10.1111/jocn.16475, PMID: 35918887

[44] Gesner E Dykes PC Zhang L Gazarian P. Documentation burden in nursing and its role in clinician burnout syndrome. Appl Clin Inform. (2022) 13:983–90. doi: 10.1055/s-0042-1757157, PMID: 36261113 PMC9581587

[45] Boateng YA Osei SA Aboh IK. Causes of burnout syndrome and coping strategies among high dependency unit nurses of an institution in the greater Accra region of Ghana. Nurs Open. (2021) 8:3334–9. doi: 10.1002/nop2.1052, PMID: 34468085 PMC8510731

[46] Salem EA Ebrahem SM. Psychosocial work environment and oxidative stress among nurses. J Occup Health. (2018) 60:182–91. doi: 10.1539/joh.17-0186-OA, PMID: 29311439 PMC5886886

[47] Dall’Ora C Griffiths P Ball J. Association of 12 h shifts and nurses’ job satisfaction, burnout and intention to leave: findings from a cross-sectional study of 12 European countries. BMJ Open. (2015) 5:e008331. doi: 10.1136/bmjopen-2015-008331, PMID: 26359284 PMC4577950

[48] Vidotti V Ribeiro RP Galdino MJQ Martins JT. Burnout syndrome and shift work among the nursing staff. Rev Lat Am Enfermagem. (2018) 26:e3022. doi: 10.1590/1518-8345.2550.3022, PMID: 30110099 PMC6091368

[49] Cañadas-De la Fuente GA Ortega E Ramirez-Baena L. Gender, marital status, and children as risk factors for burnout in nurses: a Meta-analytic study. Int J Environ Res Public Health. (2018) 15:2102. doi: 10.3390/ijerph15102102, PMID: 30257449 PMC6209972

[50] Ezenwaji IO Eseadi C Okide CC. Work-related stress, burnout, and related sociodemographic factors among nurses: implications for administrators, research, and policy. Medicine. (2019) 98:e13889. doi: 10.1097/MD.0000000000013889, PMID: 30653094 PMC6370177

[51] Chmura RM Bielemuk A Samujło B Strankowska M. Wpływ czynników środowiska pracy i relacji społecznych na występowanie zespołu wypalenia zawodowego wśród pielęgniarek In: Krajewska-Kułak E Szczepański M Łukaszuk C Lewko J, editors. Problemy terapeutyczno-pielęgnacyjne od poczęcia do starości. Białystok: Akademia Medyczna w Białymstoku, Wydział Pielęgniarstwa i Ochrony Zdrowia (2007). 302–10.

[52] Freeborn D. Satisfaction. Commitment, and psychological well-being among HMO physicians. West J Med. (2008) 174:13–8. doi: 10.1136/ewjm.174.1.13, PMID: 11154654 PMC1071220

