# Supplementary material for: Factors influencing burnout, stress levels, and coping strategies among nursing staff in intensive care units
Source: Front Public Health. 2025 May 22;13:1530353. doi: 10.3389/fpubh.2025.1530353 (PMC12139669; doi:10.3389/fpubh.2025.1530353)
Supplement: Supplementary file 1 [file Data_Sheet_1.docx]

**Table S1. General Burnout Index**

| **N** | **Mean** | **SD** | **Median** | **Min** | **Max** | **Q1** | **Q3** |
| --- | --- | --- | --- | --- | --- | --- | --- |
| 282 | 39,78 | 20,7 | 38,43 | 0 | 93,33 | 25,65 | 54,37 |

### *Abbreviations: N, number of participants; SD, standard deviation; Min, minimum; Max, maximum; Q1, first quartile; Q3, third quartile.*

**Table S2. MBI Subscales**

| **Podskale MBI** | **N** | **Mean** | **SD** | **Median** | **Min** | **Max** | **Q1** | **Q3** |
| --- | --- | --- | --- | --- | --- | --- | --- | --- |
| Emotional Exhaustion | 282 | 53,03 | 31,65 | 55,56 | 0 | 100 | 22,22 | 77,78 |
| Depersonalization | 282 | 39,79 | 27,85 | 40 | 0 | 100 | 20 | 60 |
| Lack of professional accomplishment | 282 | 26,51 | 25,33 | 25 | 0 | 100 | 0 | 50 |

** statistically significant relationship (p<0.05), p - test Kruskal-Wallis test + post-hoc analysis (Dunn’s test)
Abbreviations: N, number of participants; SD, standard deviation; Min, minimum; Max, maximum; Q1, first quartile; Q3, third quartile.*

**Table S3. Results of the PSS-10 Questionnaire Analysis.**

| **PSS-10 Score** | **Interpretation** | **N** | **%** |
| --- | --- | --- | --- |
| 0-13 | Low stress level | 56 | 19,86% |
| 14-19 | Moderate stress level | 104 | 36,88% |
| Powyżej 19 | High stress level | 122 | 43,26% |
